# The efficacy and safety of cangrelor in single vessel vs multivessel percutaneous coronary intervention: Insights from CHAMPION PHOENIX

**DOI:** 10.1002/clc.23221

**Published:** 2019-06-29

**Authors:** Celina M. Yong, Vandana Sundaram, Freddy Abnousi, Christoph B. Olivier, Jaden Yang, Gregg W. Stone, Philippe G. Steg, C. Michael Gibson, Christian W. Hamm, Matthew J. Price, Efthymios N. Deliargyris, Jayne Prats, Harvey D. White, Robert A. Harrington, Deepak L. Bhatt, Kenneth W. Mahaffey, on behalf of the CHAMPION PHOENIX Investigators

**Affiliations:** ^1^ Division of Cardiology, Veterans Affairs Palo Alto Healthcare System Palo Alto California; ^2^ Division of Cardiovascular Medicine Stanford University School of Medicine Stanford California; ^3^ Quantitative Sciences Unit, Department of Medicine Stanford University School of Medicine Stanford California; ^4^ Stanford Center for Clinical Research (SCCR) Department of Medicine, Stanford University School of Medicine Stanford California; ^5^ Department of Cardiology and Angiology I Heart Center Freiburg University, Faculty of Medicine, University of Freiburg Freiburg Germany; ^6^ Cardiovascular Research Foundation Columbia University Medical Center New York New York; ^7^ DHU (Département Hospitalo‐Universitaire)‐FIRE (Fibrosis, Inflammation, REmodelling), Hôpital Bichat, AP‐HPb (Assistance Publique‐Hôpitaux de Paris) Université Paris‐Diderot, Sorbonne‐Paris Cité, and FACT (French Alliance for Cardiovascular clinical Trials), an F‐CRIN network, INSERM U‐1148 Paris France; ^8^ NLHI, ICMS Royal Brompton Hospital, Imperial College London UK; ^9^ Beth Israel Deaconess Medical Center, Division of Cardiology Harvard Medical School, Boston Boston Massachusetts; ^10^ Kerckhoff Heart and Thorax Center Bad Nauheim Germany; ^11^ Scripps Clinic and Scripps Translational Science Institute La Jolla California; ^12^ Science and Strategy Consulting Group Basking Ridge New Jersey; ^13^ Elysis Carlisle Massachusetts; ^14^ Auckland City Hospital University of Auckland Auckland New Zealand; ^15^ Brigham and Women's Hospital Heart & Vascular Center Harvard Medical School Boston Massachusetts

**Keywords:** cangrelor, clopidogrel, multivessel percutaneous coronary intervention

## Abstract

**Background:**

The intravenous, rapidly acting P2Y12 inhibitor cangrelor reduces the rate of ischemic events during PCI with no significant increase in severe bleeding. However, the efficacy and safety of cangrelor compared with clopidogrel in patients treated with single vessel (SV)‐percutaneous coronary intervention (PCI) or multivessel (MV)‐PCI remains unexplored.

**Methods:**

We studied the modified intention‐to‐treat population of patients from the CHAMPION PHOENIX trial who were randomized to either cangrelor or clopidogrel. We used logistic regression and propensity score matching to evaluate the effect of cangrelor compared with clopidogrel on the primary efficacy outcome (composite of death, myocardial infarction, ischemia‐driven revascularization, or stent thrombosis) at 48 hours. The safety outcome was moderate or severe Global Utilization of Streptokinase and tPA for Occluded Arteries bleeding at 48 hours.

**Hypothesis:**

Cangrelor is as efficacious and safe as clopidogrel in both SV and MV PCI.

**Results:**

Among 10 854 patients, 9204 (85%) underwent SV‐ and 1650 (15%) MV‐PCI. After adjustment, cangrelor was associated with similar reductions vs clopidogrel in the primary efficacy outcome in patients undergoing SV‐PCI (4.5% vs 5.2%; odds ratio [OR] 0.81 [0.66‐0.98]) or MV‐PCI (6.1% vs 9.8%, OR 0.59 [0.41‐0.85]; Pint 0.14). Similar results were observed after propensity score matching (SV‐PCI: 5.5% vs 5.9%, OR 0.93 [0.74‐1.18]; MV‐PCI: 6.2% vs 8.9%, OR 0.67 [0.44‐1.01]; Pint 0.17). There was no evidence of heterogeneity in the treatment effect of cangrelor compared with clopidogrel for the safety outcome.

**Conclusions:**

In patients undergoing SV‐ or MV‐PCI, cangrelor was associated with similar relative risk reductions in ischemic complications and no increased risk of significant bleeding compared with clopidogrel, which highlights the expanding repertoire of options for use in complex PCI.

**Abbreviations**
IDR: ischemia‐driven revascularization
MI: myocardial infarction
mITT: modified intention to treat
MV‐PCI: multivessel percutaneous coronary intervention
OR: odds ratio
ST: stent thrombosis
SV‐PCI: single vessel percutaneous coronary intervention

## 1 INTRODUCTION

Rapid advances in percutaneous coronary intervention (PCI) tools and techniques are allowing operators to tackle progressively more challenging coronary revascularization procedures via a percutaneous approach. Notably, there has been an increase of multivessel (MV)‐PCI to address MV‐coronary disease as an alternative to coronary artery bypass grafting.^1^ However, MV‐PCI is associated with increased complexity and risk.^2^, ^3^ To mitigate periprocedural ischemic PCI complications, adjuvant pharmacotherapy has evolved.

The CHAMPION PHOENIX trial evaluated the efficacy and safety of cangrelor compared with clopidogrel among patients undergoing PCI for indications ranging from stable angina to all forms of acute coronary syndrome. Cangrelor reduced the rate of ischemic events at 48 hours compared with clopidogrel, without a significant increase in severe bleeding.^4^ The efficacy and safety of cangrelor compared with clopidogrel was similar in patients with single vessel (SV)‐disease and in patients with MV‐disease.^5^

However, the risks and benefits of MV‐PCI as a revascularization strategy in combination with either cangrelor or clopidogrel are still unknown. As such, we aimed to evaluate the efficacy and safety of cangrelor compared with clopidogrel among patients actually treated with SV‐PCI or with MV‐PCI in the CHAMPION PHOENIX trial.

## 2 METHODS

The rationale and design of the CHAMPION PHOENIX trial have been detailed previously^6^ and are summarized briefly here.

### 2.1 Participants

The CHAMPION PHOENIX trial included men and women aged 18 or older who required PCI for stable angina and acute coronary syndrome. For this study, we included patients enrolled in the CHAMPION PHOENIX trial who underwent SV‐ or MV‐index PCI as defined using core angiographic data. Patients in whom procedures were performed in more than one vessel (left main (LM), left anterior descending (LAD), left circumflex (LCX), right coronary artery (RCA)) were defined as having MV‐PCI; patients in whom procedures were performed in only one vessel (whether or not they had multivessel disease) were defined as having SV‐PCI. If a procedure was staged, it was only considered MV‐PCI if multiple vessels were treated in a single setting. Patients who had LM PCI were considered to have MV‐PCI. A central clinical events committee adjudicated all angiographic data.

### 2.2 Study Treatment

Prior to PCI but after angiography, patients were randomized to receive either cangrelor or clopidogrel in a double‐dummy, double‐blind manner. Patients received either a cangrelor infusion (initial bolus 30 mcg/kg) plus placebo capsules or placebo followed by a loading dose of clopidogrel (300 or 600 mg, either before or after PCI, per site investigator). After loading, patients in the cangrelor arm received a 4 mcg/kg/min infusion of cangrelor for at least 2 hours or until the procedure was complete, whichever was longer. All patients received aspirin (75 to 325 mg). All patients received 75 mg clopidogrel as maintenance therapy. Periprocedural use of bivalirudin, unfractionated heparin, low molecular weight heparin, or fondaparinux was allowed at the discretion of the site investigator, with a glycoprotein IIb/IIIa inhibitor as an option only for rescue therapy.

### 2.3 Efficacy outcomes

We considered the same primary efficacy outcome for this analysis as in CHAMPION PHOENIX, defined as the composite of death from any cause, myocardial infarction (MI), ischemia‐driven revascularization (IDR), or stent thrombosis (ST) at 48 hours after randomization. We also evaluated the same composite outcome at 30 days as the secondary efficacy analysis. A central clinical event committee adjudicated all suspected events of the efficacy outcome.^5^

### 2.4 Safety outcomes

The primary safety outcome for the purposes of this analysis was defined as moderate or severe Global Utilization of Streptokinase and tPA for Occluded Arteries bleeding at 48 hours after randomization (due to the small number of events of the pre‐specified primary safety endpoint of severe Global Utilization of Streptokinase and tPA for Occluded Arteries bleeding in the original trial). According to prespecified definitions, bleeding end points were reported by the blinded site investigators and not centrally adjudicated.^7^

### 2.5 Statistical analysis

The analyses were conducted in the prespecified modified intention‐to‐treat (mITT) population, which included all randomized patients who underwent PCI and received at least one dose of study drug.

Univariable analysis was used to compare demographic and clinical characteristics between the two procedure groups (SV‐ vs MV‐PCI). We used logistic regression techniques to assess efficacy at 48 hours and 30 days. Our primary analysis was logistic regression of outcome on treatment group, procedure group (SV‐ or MV‐PCI), the interaction between treatment group and procedure group, and with adjustment for selected characteristics in the original cohort. We selected baseline demographic and clinical characteristics that were significantly different (two‐sided significance level of 0.01) between participants undergoing SV‐ vs MV‐PCI (sex, history of diabetes, access, bare‐metal stent, drug‐eluting stent, clopidogrel loading dose (600 vs 300), region, clinical presentation (stable angina vs acute coronary syndrome), bifurcation treated), and characteristics that were deemed clinically relevant (time to PCI procedure from study drug administration, lesion calcification, lesion tortuosity, and PCI duration).

We performed a propensity analysis to minimize variance in baseline demographic and clinical characteristics of patients in the two procedure groups, since MV or SV‐PCI was a post randomization event. We estimated the propensity score using logistic regression where we regressed MV‐PCI on sex, history of diabetes, bifurcation treated, presentation, and clinically relevant characteristics listed above; we did not match on access, use of bare metal or drug‐eluting stent, or periprocedural clopidogrel dose because they were significantly associated with region. Patients were matched in a 1:4 ratio on propensity score; we did an exact match for region and used a 5% caliper matching for propensity score for the other variables. Because some of the clinical variables of interest were highly region‐dependent, we used an exact match for region to achieve more comparable groups. We conducted a mixed effects logistic regression model on the 48‐hour and 30‐day composite outcomes using the matched cohort; the analysis was clustered on the match strata. We regressed outcome on treatment group, procedure group (SV‐ vs MV‐PCI), and the interaction between treatment group and procedure group. Analyses were conducted using SAS software, version 9.3 (SAS Institute, Cary, North Carolina) and R (2014 version, R Core Team, Vienna, Austria).

## 3 RESULTS

### 3.1 Baseline characteristics

Of the 10 942 patients from the mITT population, SV‐ vs MV‐PCI status could be assessed by the core angiography data in 10 854 patients. Of those patients, 9204 (85%; 4580 cangrelor and 4624 clopidogrel) underwent SV‐PCI and 1650 (15%, 846 cangrelor and 804 clopidogrel) underwent MV‐PCI. Among patients who underwent SV‐PCI, 56% had SV‐disease and 44% had MV‐disease.

Patients who were treated with MV‐PCI were more likely to be male (75% vs 71%), be enrolled in the United States (42% vs 37%), have diabetes (32% vs 27%), present with either stable angina (62% vs 56%) or non‐ST elevation myocardial infarction (23% vs 19%), undergo femoral access (77% vs 73%), be treated with a drug‐eluting stent (61% vs 55%), and have a longer PCI duration (median 27 [interquartile range 16‐40] minutes vs 15 [9‐27] minutes) (Tables 1 and 2).

**Table 1:** Baseline characteristics by single vs multivessel PCI and medication treatment group

			Single vessel PCI		Multivessel PCI	
	Single vessel PCI	Multivessel PCI	Cangrelor	Clopidogrel	Cangrelor	Clopidogrel
	(N = 9204)	(N = 1650)	(N = 4580)	(N = 4624)	(N = 846)	(N = 804)
Age (median, IQR)	64.00 [56.00, 72.00]	65.00 [57.00, 72.75]	64.00 [56.00, 72.00]	64.00 [56.00, 72.00]	65.00 [57.00, 72.00]	64.00 [57.00, 73.00]
Female, N (%)	2621 (28.5)	408 (24.7)	1341 (29.3)	1280 (27.7)	204 (24.1)	204 (25.4)
BMI (median, IQR)	28.37 [25.47, 31.79]	28.40 [25.85, 31.96]	28.38 [25.47, 31.79]	28.35 [25.49, 31.77]	28.24 [25.71, 31.98]	28.71 [26.09, 31.92]
Weight (median, IQR)	84.00 [73.00, 95.30]	84.45 [75.00, 96.00]	84.00 [73.00, 95.00]	84.00 [73.00, 95.82]	84.00 [74.00, 95.50]	85.00 [75.00, 96.40]
White race	8605 (93.5)	1562 (94.7)	4288 (93.6)	4317 (93.4)	800 (94.6)	762 (94.8)
**Medical history (%)**						
DM2	2508 (27.3)	522 (31.7)	1245 (27.2)	1263 (27.4)	261 (30.9)	261 (32.5)
Current Tobacco use	2597 (28.9)	418 (26.1)	1269 (28.4)	1328 (29.4)	213 (26.1)	205 (26.1)
Hyperlipidemia	5615 (68.5)	1065 (72.7)	2787 (68.4)	2828 (68.7)	566 (74.6)	499 (70.8)
Hypertension	7314 (79.7)	1325 (80.3)	3664 (80.2)	3650 (79.2)	679 (80.3)	646 (80.3)
Prior Stroke/transient ischemic attack (TIA)	430 (4.7)	82 (5.0)	227 (5.0)	203 (4.4)	42 (5.0)	40 (5.0)
Prior MI	1877 (20.5)	373 (22.7)	899 (19.7)	978 (21.3)	182 (21.6)	191 (23.9)
Prior percutaneous transluminal coronary angioplasty/percutaneous coronary intervention (PTCA/PCI)	2133 (23.2)	457 (27.7)	1031 (22.6)	1102 (23.9)	232 (27.4)	225 (28.0)
Prior coronary artery bypass graft (CABG)	891 (9.7)	184 (11.2)	478 (10.4)	413 (8.9)	99 (11.7)	85 (10.6)
congestive heart failure (CHF)	932 (10.1)	203 (12.3)	450 (9.8)	482 (10.5)	101 (12.0)	102 (12.7)
peripheral artery disease (PAD)	676 (7.4)	155 (9.5)	352 (7.8)	324 (7.1)	95 (11.4)	60 (7.5)
**Region (%)**						
US	3388 (36.8)	700 (42.4)	1691 (36.9)	1697 (36.7)	352 (41.6)	348 (43.3)
**Presentation (%)**						
Non‐ST Elevation Myocardial Infarction (NSTEMI)	1779 (19.3)	386 (23.4)	849 (18.5)	930 (20.1)	216 (25.5)	170 (21.1)
Stable angina	5104 (55.5)	1018 (61.7)	2603 (56.8)	2501 (54.1)	506 (59.8)	512 (63.7)
ST Elevation Myocardial Infarction (STEMI)	1798 (19.5)	151 (9.2)	863 (18.8)	935 (20.2)	74 (8.7)	77 (9.6)
Unstable angina	523 (5.7)	95 (5.8)	265 (5.8)	258 (5.6)	50 (5.9)	45 (5.6)
**Access (%)**						
Brachial access	21 (0.2)	2 (0.1)	7 (0.2)	14 (0.3)	2 (0.2)	0 (0.0)
Femoral access	6709 (72.9)	1277 (77.4)	3351 (73.2)	3358 (72.6)	661 (78.1)	616 (76.6)
Radial access	2474 (26.9)	371 (22.5)	1222 (26.7)	1252 (27.1)	183 (21.6)	188 (23.4)
**Peri‐procedural medications (%)**						
Aspirin	8683 (94.4)	1542 (93.5)	4323 (94.4)	4360 (94.4)	796 (94.2)	746 (92.8)
Unfractionated Heparin	7161 (77.8)	1306 (79.2)	3573 (78.0)	3588 (77.6)	656 (77.5)	650 (80.8)
Low molecular weight heparin	1249 (13.6)	232 (14.1)	610 (13.3)	639 (13.8)	120 (14.2)	112 (13.9)
Clopidogrel 600	6953 (75.5)	1127 (68.3)	3455 (75.4)	3498 (75.6)	584 (69.0)	543 (67.5)
Clopidogrel 300	2251 (24.5)	523 (31.7)	1125 (24.6)	1326 (24.4)	262 (31.0)	261 (32.5)
Glycoprotein IIb/IIIa inhibitor						
Unplanned	285 (83.3)	34 (89.5)	114 (82.0)	171 (84.2)	13 (92.9)	21 (87.5)
Bivalirudin	2108 (22.9)	405 (24.5)	1036 (22.6)	1072 (23.2)	212 (25.1)	193 (24.0)
Fondaparinux	227 (2.5)	64 (3.9)	118 (2.6)	109 (2.4)	38 (4.5)	26 (3.2)

Abbreviations: BMI, body mass index; IQR, interquartile range; MI, myocardial infarction; PCI, percutaneous coronary intervention.

**Table 2:** Procedural and anatomic characteristics by single vs multivessel PCI and medication treatment group

			Single vessel PCI		Multivessel PCI	
	Single vessel PCI	Multivessel PCI	Cangrelor	Clopidogrel	Cangrelor	Clopidogrel
	(N = 9204)	(N = 1650)	(N = 4580)	(N = 4624)	(N = 846)	(N = 804)
**Intervention**						
BMS (Bare‐metal stent)	3825 (41.6)	760 (46.1)	1881 (41.1)	1944 (42.0)	393 (46.5)	367 (45.6)
DES (Drug‐eluting stent)	5058 (55.0)	1006 (61.0)	2533 (55.3)	2525 (54.6)	518 (61.2)	488 (60.7)
Balloon‐Angioplasty	486 (100.0)	77 (100.0)	244 (5.3)	242 (5.2)	46 (5.4)	31 (3.9)
Number of stents (any)	1.00 [1.00, 2.00]	2.00 [2.00, 3.00]	1.00 [1.00, 2.00]	1.00 [1.00, 2.00]	2.00 [2.00, 3.00]	2.00 [2.00, 3.00]
Number of Drug‐eluting Stents	1.00 [1.00, 1.00]	2.00 [2.00, 3.00]	1.00 [1.00, 1.00]	1.00 [1.00, 1.00]	2.00 [2.00, 3.00]	2.00 [2.00, 2.50]
Number of Non Drug‐ eluting Stents	1.00 [1.00, 2.00]	2.00 [2.00, 3.00]	1.00 [1.00, 2.00]	1.00 [1.00, 2.00]	2.00 [2.00, 3.00]	2.00 [2.00, 3.00]
Length of stented area (all stents) (median, IQR)	23.00 [15.00, 31.00]	40.00 [28.00, 58.00]	23.00 [15.00, 31.00]	23.00 [15.00, 32.00]	39.00 [27.00, 56.00]	41.00 [28.00, 59.00]
Length of Drug‐eluting Stents (median, IQR)	20.00 [15.00, 28.00]	37.00 [26.00, 50.00]	20.00 [15.00, 28.00]	20.00 [15.00, 28.00]	37.00 [26.00, 51.00]	36.00 [26.00, 48.00]
Length of Non Drug‐eluting Stents (median, IQR)	22.00 [15.00, 30.00]	41.00 [30.00, 57.00]	22.00 [15.00, 30.00]	22.00 [15.00, 30.00]	41.00 [30.00, 56.00]	41.00 [30.00, 58.00]
Time from study drug administration to start of PCI min (median, IQR)	4.00 [2.00, 8.00]	3.00 [1.00, 7.00]	4.00 [2.00, 8.00]	4.00 [2.00, 8.00]	3.00 [1.00, 7.00]	3.00 [1.00, 7.00]
Duration of PCI (median, IQR)	15.00 [9.00, 27.00]	27.00 [16.00, 40.00]	16.00 [9.00, 27.50]	15.00 [9.00, 27.00]	27.00 [17.00, 38.75]	27.00 [15.00, 40.00]
**Type of vessel**						
LM	94 (1.0)	181 (11.0)	56 (1.2)	38 (0.8)	92 (10.9)	89 (11.1)
LAD	4191 (45.5)	1189 (72.1)	2105 (46.0)	2086 (45.1)	619 (73.2)	570 (70.9)
LCX	2096 (22.8)	1036 (62.8)	1036 (22.6)	1060 (22.9)	533 (63.0)	503 (62.6)
RCA	2966 (32.2)	912 (55.3)	1471 (32.1)	1495 (32.3)	458 (54.1)	454 (56.5)
Bifurcation treated	55 (0.6)	76 (4.6)	36 (0.8)	19 (0.4)	49 (5.8)	27 (3.4)
Lesion tortuosity (moderate)	189 (2.1)	37 (2.2)	99 (2.2)	90 (1.9)	24 (2.8)	13 (1.6)
Lesion tortuosity (severe)	48 (0.5)	7 (0.4)	25 (0.5)	23 (0.5)	2 (0.2)	5 (0.6)
Calcification (moderate)	1725 (18.7)	281 (17.0)	846 (18.5)	879 (19.0)	149 (17.6)	132 (16.4)
Calcification (severe)	460 (5.0)	80 (4.8)	242 (5.3)	218 (4.7)	38 (4.5)	42 (5.2)

Abbreviations: IQR, interquartile range; PCI, percutaneous coronary intervention.

### 3.2 48‐hour outcomes

**Figure 1:**
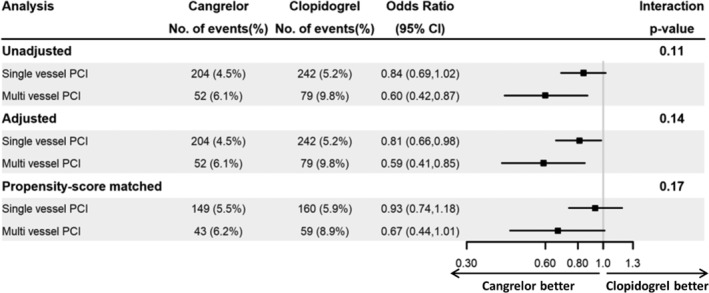
Logistic regression results for 48‐hour composite outcome. There was no heterogeneity in treatment effect associated with cangrelor compared with clopidogrel observed for the 48‐hour primary composite efficacy outcome for patients treated with SV‐PCI vs MV‐PCI patients in the unadjusted, adjusted, or propensity‐score matched analyses. Abbreviations: CI, confidence interval; MV, multivessel; No, number; PCI, percutaneous coronary intervention; SV, single vessel

In the unadjusted analysis, there was no heterogeneity in treatment effect associated with cangrelor compared with clopidogrel observed for the 48‐hour primary composite efficacy outcome for patients treated with SV‐PCI (4.5% vs 5.2%; odds ratio [OR] 0.84, 95% confidence interval [CI] 0.69‐1.02) vs MV‐PCI (6.1% vs 9.8%; OR 0.60, 95% CI [0.42‐0.87]; interaction *P*‐value .11) (Figure 1). Similar results were observed after adjustment (SV‐PCI OR 0.81, 95% CI [0.66‐0.98]), MV‐PCI (OR 0.59, 95% CI [0.41‐0.85]; interaction *P*‐value .14). We matched 1357 patients in the MV‐PCI group to 5428 patients in the SV‐PCI group through propensity scores. For the 48‐hour primary composite efficacy outcome, patients in both the SV‐ and MV‐PCI groups had a similar decreased odds of the 48‐hour primary composite efficacy outcome associated with cangrelor compared with clopidogrel (SV‐PCI: 5.5% vs 5.9%, OR 0.93 [0.74‐1.18]; MV‐PCI: 6.2% vs 8.9%, OR 0.67 [0.44‐1.01]; interaction *P* value .17).

### 3.3 30 day outcomes

**Figure 2:**
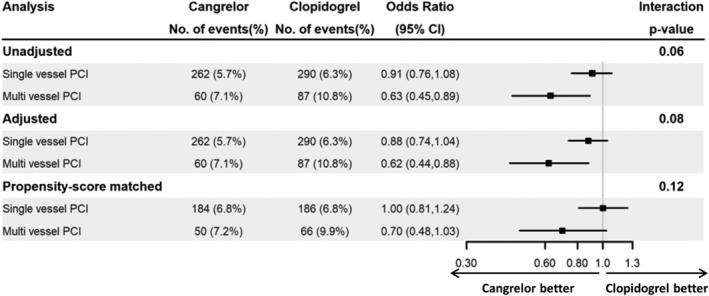
Logistic regression results for 30‐day composite outcome. No significant difference was observed in the treatment effect associated with cangrelor compared with clopidogrel for the 30‐day composite efficacy outcome in participants treated with SV‐ vs MV‐PCI in the unadjusted, adjusted, or propensity‐score matched analyses. Abbreviations: CI, confidence interval; MV, multivessel; No, number; PCI, percutaneous coronary intervention; SV, single vessel

No significant difference was observed in the treatment effect associated with cangrelor compared with clopidogrel for the 30‐day composite efficacy outcome in participants treated with SV‐ vs MV‐PCI in the unadjusted analysis (interaction *P*‐value .06) or adjusted analysis (interaction *P*‐value .08, Figure 2).

In the matched cohorts, there was no significant evidence of heterogeneity in the treatment effect for patients in the SV‐ and MV‐PCI groups for the 30‐day composite efficacy outcome associated with cangrelor compared with clopidogrel (SV‐PCI: OR: 1.00 [95% CI: 0.81‐1.24]; MV‐PCI: 0.70 [95% CI: 0.48‐1.03], interaction *P*‐value .12) (Figure 2).

### 3.4 Safety Outcomes

**Figure 3:**
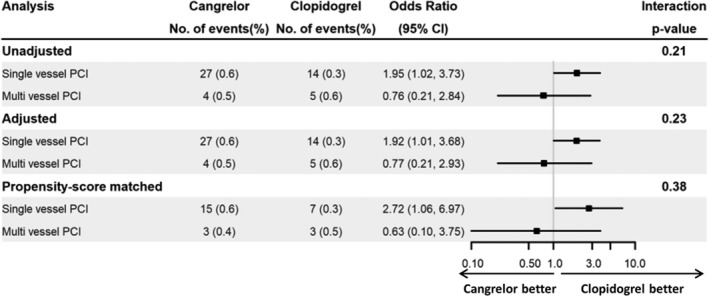
Safety outcomes (moderate/severe global utilization of streptokinase and tPA for occluded arteries bleeding) at 48 hours. Rates of moderate/severe bleeding were low (<1%) with no heterogeneity in the treatment effect associated with cangrelor compared with clopidogrel for SV‐ vs MV‐PCI in the unadjusted, adjusted, or propensity‐score matched analyses. Abbreviations: CI, confidence interval; MV, multivessel; No, number; PCI, percutaneous coronary intervention; SV, single vessel

For moderate/severe Global Utilization of Streptokinase and tPA for Occluded Arteries bleeding, rates were low (<1%) with no heterogeneity in the treatment effect associated with cangrelor compared with clopidogrel for SV‐ vs MV‐PCI either in the unadjusted, the adjusted, or the propensity score matched analysis (Figure 3).

## 4 DISCUSSION

This study is the first to provide two notable findings: (a) cangrelor was associated with a similar reduction in risk of death/MI/IDR/ST compared with clopidogrel among patients treated with SV‐ vs MV‐PCI, with consistent results after adjustment and propensity matching, (b) cangrelor was not associated with an increase in the risk of moderate or severe bleeding compared with clopidogrel among patients treated with SV‐ vs MV‐PCI.

Analysis of trends over time clearly demonstrate a dramatic increase in the use of MV‐PCI,^8^, ^9^ emphasizing the importance of establishing the relative efficacy and safety of novel antiplatelet agents in both SV‐ and MV‐PCI procedures. It is plausible that cangrelor might actually offer a benefit over clopidogrel in many MV‐PCI scenarios, which is supported by the numerical trend in absolute risk reduction for the composite outcome among MV‐PCI patients in this study. However, given the nonsignificant *P* value for the interaction, this study demonstrates that cangrelor was associated with similar efficacy in both procedural strategies. This may inform the selection of a particular antiplatelet medication strategy at the start of a case, which is often before final decisions about degree of revascularization are necessarily made. Additionally, in combination with recent findings of the benefit of cangrelor in the treatment of lesions with high risk features,^10^ these results suggest an expanded repertoire of clinical scenarios in which cangrelor may be the antiplatelet agent of choice.

While recent studies suggest that MV‐PCI is safe,^9^, ^11^, ^12^, ^13^, ^14^ rigorous data from post hoc analyses of randomized controlled trials still demonstrate an increase of death, MI, or major adverse cardiac events (MACE) risks with MV‐PCI, suggesting particularly important treatment scenarios in which cangrelor may provide value.^15^ For example, higher peri‐procedural risk with MV‐PCI portends a greater likelihood that conversion to emergent open coronary artery bypass surgery may be necessary as a bailout—one of the most important times that the short half‐life of cangrelor would be advantageous. Increasingly, hybrid surgical‐PCI techniques are being chosen for the treatment of MV coronary artery disease. In these cases, cangrelor may allow for more closely timed surgical and PCI procedures that may save hospital length of stay and decrease bleeding; further study of cangrelor in this domain should be explored.

While prior data suggest higher post‐PCI bleeding risk among patients with multivessel coronary artery disease (CAD),^16^ our study demonstrated no increased risk of moderate to severe Global Utilization of Streptokinase and tPA for Occluded Arteries bleeding among patients who underwent SV‐ and MV‐PCI. Qualitatively, there was a trend towards lower bleeding among MV‐PCI patients treated with cangrelor, however, the odds ratios showed no significant difference. Note that these findings are tempered by the (a) overall low event rates, and (b) potential treatment effect: patients with bleeding complications during their first PCI may not proceed to a second PCI.

There are limitations to this study. First, this was not a prespecified subgroup analysis. PCI was a postrandomization variable; as such, while we performed propensity matching to attempt to account for numerous potential confounders, it is possible that other confounders persist. For example, if an ischemic complication occurred during the first PCI, the decision might be made to not proceed to a second PCI. As a result, we could be underestimating the treatment effect in planned MV‐PCI. Secondly, while extensive core angiographic data did provide details on tortuosity and calcification, some measures of the complexity of PCI or the overall clinical risk of each procedure (ranging from stable angina to ST elevation Myocardial Infarction presentation) were not systematically assessed. Fractional flow reserve was not used to assess the functional significance of disease in each vessel, which has been shown to differentiate necessity of intervention better than angiographic appearance alone.^17^ Additionally, while 45% of patients who received SV‐PCI had MV‐disease, we did not systematically collect information why exactly MV‐PCI was not pursued in those patients and the degree to which this may have resulted in incomplete revascularization.^18^ As such, our results reflect a cumulative summary of outcomes rather than the likely heterogeneity within each PCI procedure group.

These findings among a contemporary cohort of patients across the full spectrum of acute coronary syndrome suggest that cangrelor compared with clopidogrel is associated with a similar reduction in the composite of death, MI, IDR, and ST at 48 hours in patients undergoing PCI with consistent results in patients treated with SV‐ and MV‐PCI without an associated increased risk of severe bleeding.

## CONFLICT OF INTEREST

Drs Celina M. Yong and Freddy Abnousi have no disclosures.

Dr Christian W. Hamm discloses honoraria from Abbott, AstraZeneca, Bayer, Boehringer Ingelheim, Daiichi Sankyo, SanofiAventis, Pfizer, and The Medicines Company.

Dr Philippe G. Steg discloses the following relationships: research grant (to INSERM U1148) from Sanofi and Servier, and speaking or consulting fees from Amarin, AstraZeneca, Bayer, Boehringer‐Ingelheim, Bristol‐Myer Squibb, CSL‐Behring, Daiichi‐Sankyo, GlaxoSmithKline, Janssen, Lilly, Merck, Novartis, Pfizer, Regeneron, Roche, Sanofi, Servier, and The Medicines Company; he also owns stocks from Aterovax.

Dr C. M. Gibson discloses modest consulting for The Medicines Company.

Dr Harvey D. White discloses the following relationships: honoraria from AstraZeneca, and research funding from Sanofi‐Aventis, Eli Lilly, National Health Institute, Glaxo Smith Kline, Merck Sharpe & Dohme, and AstraZeneca.

Dr Matthew J. Price discloses consulting and speaker's honoraria from The Medicines Company and AstraZeneca, and consulting honoraria from Merck, Boeringher Ingleheim, Terumo, Boston Scientific, Medtronic, and St Jude Medical.

Dr Robert A. Harrington discloses research grants/contracts from the National Heart Lung and Blood Institute, Duke, AstraZeneca, CSL‐Behring, Glaxo Smith Kline, Merck, Portola, Regado, Sanofi‐Aventis, and The Medicines Company, and consulting/advisory for Adverse Events, Amgen, Element Science, Gilead, Merck, MyoKardia, The Medicines Company, VidaHealth, and WebMD.

Dr Deepak L. Bhatt discloses the following relationships—Advisory Board: Cardax, Elsevier Practice Update Cardiology, Medscape Cardiology, Regado Biosciences; Board of Directors: Boston VA Research Institute, Society of Cardiovascular Patient Care, TobeSoft; Chair: American Heart Association Quality Oversight Committee; Data Monitoring Committees: Baim Institute for Clinical Research (formerly Harvard Clinical Research Institute, for the PORTICO trial, funded by St. Jude Medical, now Abbott), Cleveland Clinic, Duke Clinical Research Institute, Mayo Clinic, Mount Sinai School of Medicine (for the ENVISAGE trial, funded by Daiichi Sankyo), Population Health Research Institute; Honoraria: American College of Cardiology (Senior Associate Editor, Clinical Trials and News, ACC.org; Vice‐Chair, ACC Accreditation Committee), Baim Institute for Clinical Research (formerly Harvard Clinical Research Institute; RE‐DUAL PCI clinical trial steering committee funded by Boehringer Ingelheim), Belvoir Publications (Editor in Chief, Harvard Heart Letter), Duke Clinical Research Institute (clinical trial steering committees), HMP Global (Editor in Chief, Journal of Invasive Cardiology), Journal of the American College of Cardiology (Guest Editor; Associate Editor), Population Health Research Institute (for the COMPASS operations committee, publications committee, steering committee, and USA national co‐leader, funded by Bayer), Slack Publications (Chief Medical Editor, Cardiology Today's Intervention), Society of Cardiovascular Patient Care (Secretary/Treasurer), WebMD (CME steering committees); Other: Clinical Cardiology (Deputy Editor), NCDR‐ACTION Registry Steering Committee (Chair), VA CART Research and Publications Committee (Chair); Research Funding: Abbott, Amarin, Amgen, AstraZeneca, Bayer, Boehringer Ingelheim, Bristol‐Myers Squibb, Chiesi (including for his role as Co‐Chair of CHAMPION PHOENIX), Eisai, Ethicon, Forest Laboratories, Idorsia, Ironwood, Ischemix, Lilly, Medtronic, PhaseBio, Pfizer, Regeneron, Roche, Sanofi Aventis, Synaptic, The Medicines Company (including for his role as Co‐Chair of CHAMPION PHOENIX); Royalties: Elsevier (Editor, Cardiovascular Intervention: A Companion to Braunwald's Heart Disease); Site Co‐Investigator: Biotronik, Boston Scientific, St. Jude Medical (now Abbott), Svelte; Trustee: American College of Cardiology; Unfunded Research: FlowCo, Merck, Novo Nordisk, PLx Pharma, Takeda.

Dr Kenneth W. Mahaffey's financial disclosures can be viewed at http://med.stanford.edu.laneproxy.stanford.edu/profiles/kenneth‐mahaffey.

Vandana Sundaram, Manisha Desai, and Lingyao Yang have received salary support from The Medicines Company and Chiesi‐US.

Drs Efthymios N. Deliargyris and Jayne Prats were full‐time employees of The Medicines Company at the time of the study. Dr Prats receives consulting fees from Chiesi‐US, the current sponsor of cangrelor.

Dr Christoph B. Olivier reports research support from the German Research Foundation and personal fees from Bayer Vital GmbH [Conflict of interest statement added on 19 August 2019, after first online publication.]
